# Role of ASM/Cer/TXNIP signaling module in the NLRP3 inflammasome activation

**DOI:** 10.1186/s12944-021-01446-4

**Published:** 2021-02-21

**Authors:** Jianjun Jiang, Yining Shi, Jiyu Cao, Youjin Lu, Gengyun Sun, Jin Yang

**Affiliations:** 1 Department of Respiratory and Critical Care Medicine, the First Affiliated Hospital of Anhui Medical University, 218 Jixi Road, Hefei, 230022 Anhui China; 2 Department of Respiratory and Critical Care Medicine, the Second Affiliated Hospital of Anhui Medical University, 678 Furong Road, Hefei, 230601 Anhui China; 3 Department of Occupational Health and Environmental Health, School of Public Health, Anhui Medical University, 81 Meishan Road, Hefei, 230032 Anhui China

**Keywords:** Lipopolysaccharide, J774A.1 cells, THP-1 macrophages, Thioredoxin interacting protein, Acid sphingomyelinase, NOD-like receptor Protein3, Ceramide

## Abstract

**Background:**

This study aimed to explore the effects of ceramide (Cer) on NLRP3 inflammasome activation and their underlying mechanisms.

**Methods:**

Lipopolysaccharide (LPS)/adenosine triphosphate (ATP)-induced NLRP3 inflammasome activation in J774A.1 cells and THP-1 macrophages was used as an in vitro model of inflammation. Western blotting and real-time PCR (RT-PCR) were used to detect the protein and mRNA levels, respectively. IL-1β and IL-18 levels were measured by ELISA. ASM assay kit and immunofluorescence were used to detect ASM activity and Cer content.

**Results:**

Imipramine, a well-known inhibitor of ASM, significantly inhibited LPS/ATP-induced activity of ASM and the consequent accumulation of Cer. Additionally, imipramine suppressed the LPS/ATP-induced expression of thioredoxin interacting protein (TXNIP), NLRP3, caspase-1, IL-1β, and IL-18 at the protein and mRNA level. Interestingly verapamil, a TXNIP inhibitor, suppressed LPS/ATP-induced activation of TXNIP/NLRP3 inflammasome but did not affect LPS/ATP-induced ASM activation and Cer formation. TXNIP siRNA and verapamil inhibited C2-Cer-induced upregulation of TXNIP and activation of the NLRP3 inflammasome. In addition, the pretreatment of cells with sulfo-N-succinimidyl oleate (SSO), an irreversible inhibitor of the scavenger receptor CD36, blocked Cer-induced upregulation of nuclear factor-κB (NF-κB) activity, TXNIP expression, and NLRP3 inflammasome activation. Inhibition of NF-κB activation by SN50 prevented Cer-induced upregulation of TXNIP and activation of the NLRP3 inflammasome but did not affect CD36 expression.

**Conclusion:**

This study demonstrated that the ASM/Cer/TXNIP signaling pathway is involved in NLRP3 inflammasome activation. The results documented that the CD36-dependent NF-κB-TXNIP signaling pathway plays an essential role in the Cer-induced activation of NLRP3 inflammasomes in macrophages.

## Introduction

Inflammation is a local protective mechanism evoked by the exposure of cells to harmful stimuli, such as irritants, pathogens, or damaged cells. Excessive or chronic inflammation gives rise to multiple pathological complications, such as tissue damage and dysfunction, diabetes, sepsis, arteriosclerosis, Alzheimer’s disease, liver disorders, and various malignancies [1]. Commonly prescribed anti-inflammatory drugs fall under two categories: non-steroidal and steroidal. However, these drugs produce a range of side-effects, which restricts their clinical utility [2, 3]. This limitation necessitates identifying effective alternatives for current anti-inflammatory medications.

NOD-Like Receptor Protein 3 (NLRP3) inflammasome is a component of the crucial intracellular inflammatory pathway of the innate immune system. Chronic activation of the NLRP3 inflammasome culminates in the accumulation of pro-inflammatory cytokines, i.e., interleukin-1β (IL-1β) and interleukin-18 (IL-18), contributing to excessive inflammation and inflammatory disorders [4]. Unraveling the precise regulatory mechanism leading to the activation of NLRP3 inflammasome could help determine molecular drug targets to treat inflammatory diseases.

NLRP3 is activated by exogenous factors, such as pathogens, or endogenous factors, such as ceramide (Cer), an intracellular lipid metabolite [5, 6]. Recent studies demonstrated that Cer mediates the activation of NLRP3 inflammasome in multiple disorders, such as obesity, glomerular injury, acute lung injury, and Alzheimer’s disease [7, 8]. Cer is primarily synthesized through sphingolipid metabolism. Sphingomyelin hydrolysis is one of the first pathways activated during the stress response, and sphingomyelinase serves as a crucial component in the regulation of this pathway [9]. Acid sphingomyelinase (ASM) is localized in the lysosomes and can be activated by diverse stress stimuli, such as oxidative stress, lipopolysaccharide (LPS), and tumor necrosis factor-α (TNF-α). Cer is generated through the ASM-mediated hydrolysis of sphingomyelin at the cell membrane [10, 11]. Numerous studies have documented that the over-activation of the ASM/Cer pathway constitutes the underlying mechanism for the stimulus-induced inflammation [12, 13]. We have previously demonstrated that imipramine remarkably reduced the LPS-induced pulmonary inflammation in mice and increased their survival rate by suppressing the intracellular accumulation of Cer [14]. Moreover, NLRP3 shRNA suppressed the Cer-induced activation of NLRP3 inflammasome and secretion of pro-inflammatory cytokines, improving the permeability of type II alveolar epithelial cells [8]. However, further research is necessary on the participation of ASM-hydrolyzed sphingomyelin in the Cer-induced activation of NLRP3 inflammasome in response to stress stimuli. Thioredoxin-interacting protein (TXNIP) can directly interact with and activate the NLRP3 inflammasome [15]. Previous studies have identified the crucial role of the TXNIP/NLRP3 inflammasome signaling pathway in activating the NLRP3 inflammasome. However, whether Cer promotes TXNIP expression in macrophages has not been determined.

CD36 is a multifunctional receptor that binds a variety of ligands [16]. It is expressed in many cell types, including macrophages, adipocytes, endothelial cells, and epithelial cells, and plays an essential role in many different biological processes [16, 17]. In addition, CD36 can act as an independent pattern recognition receptor for various bacteria and bacterial cell wall components and can directly initiate intracellular signaling pathways leading to the release of pro-inflammatory cytokines [18]. Therefore, we investigated whether CD36 mediates the activation of TXNIP and subsequently induces the activation of NLRP3 inflammasomes in macrophages through Cer.

The main objective of this study was to determine whether ASM-derived Cer is involved in the NLRP3 inflammasome activation. We also attempted to identify the mechanism underlying the activation of the NLRP3 inflammasome, with a particular emphasis on the ASM/Cer/TXNIP signaling pathway.

## Materials and methods

### Materials

C2-Cer, LPS, adenosine 5′-triphosphate (ATP) disodium salt hydrate, SN50, verapamil, imipramine, sulfo-N-succinimidyl oleate (SSO) and phorbol 12-myristate 13-acetate (PMA) were acquired from Sigma-Aldrich (St Louis, MO, USA). Anti-NLRP3, anti-TXNIP, anti-CD36, anti-nuclear factor-κB (NF-κB) and anti-p-NF-κB antibodies were purchased from Abcam (San Francisco, CA, USA). The secondary antibody was obtained from ZSGB-BIO (Beijing, China). Anti-caspase-1, anti-β-actin, and lysis buffer were obtained from Cell Signaling Technology (Beverly, MA, USA).

### Cell culture and treatment

Murine macrophage cell line J774A.1 and human monocyte leukemia cell line (THP-1) were obtained from the University of Science and Technology of China. In the subsequent experiments, THP-1 monocytes were stimulated with PMA (100 nM) for 24 h to induce them to differentiate into adherent macrophages. The J774A.1 cells and THP-1 macrophages were randomly divided into 1) normal control group, LPS/ATP group, imipramine intervention + LPS/ATP group (cells were incubated with 1 μg/mL LPS for 4 h, treated with 10 μmol/L imipramine for 3 h, and then with 5 mM ATP for 30 min), and imipramine control group (imipramine group); 2) normal control group, LPS/ATP group, verapamil intervention + LPS/ATP group (cells were incubated with 1 μg/mL LPS for 4 h, treated with 10 μmol/L verapamil for 3 h, and then with 5 mM ATP for 30 min), and verapamil control group (verapamil group); 3) normal control group, Cer group (30 μmol/L C2-Cer), verapamil intervention + Cer group (cells were pretreated with 10 μmol/L verapamil for 3 h and co-incubated with 30 μmol/L C2-Cer 5 h, and TXNIP siRNA + Cer group (cells were transfected with TXNIP siRNA and incubated with 30 μmol/L C2-Cer for 5 h; and 4) normal control group, Cer group (30 μmol/L C2-Cer), SSO intervention + Cer group (cells were pretreated with 200 μmol/L SSO for 1 h and co-incubated with 30 μmol/L C2-Cer for 5 h), SSO control group, SN50 (NF-κB inhibitor) intervention + Cer group (cells were pretreated with 36 μmol/L SN50 for 1 h and co-incubated with 30 μmol/L C2-Cer for 5 h), and SN50 control group.

### Transfection

Control siRNA and TXNIP siRNA were synthesized by GenePharma (Shanghai, China). Cell transfection was performed using Lipofectamine® 3000 RNAiMax reagent (Invitrogen, Karlsruhe, Germany) according to the manufacturer’s instructions. The cells were collected for Western blotting and RT-PCR at 48 h post-transfection.

### Cell viability assays

The cells were treated with imipramine (0, 25, 50, 75, 100 μmol/L), C2-Cer (0, 15, 30, 45, 60 μmol/L) and verapamil (0, 25, 50, 75, 100 μmol/L) for 24 h and then incubated with MTT (Beyotime, Jiangsu, China) for 4 h. Subsequently, dimethylsulfoxide (DMSO) (Beyotime, Jiangsu, China) was added, and the absorbance was measured by the microplate reader (BioTek, Winooski, VT, USA) at 490 nm.

### Western blotting

An equal amount of protein (10–20 μg) was taken from each sample, loaded into the individual lane, and subjected to vertical SDS-PAGE electrophoresis (concentration gel voltage 50 V, 1 h, and separation gel voltage 100 V, 1.5 h). The separated proteins were electrophoretically transferred at 200 mA for 3.5 h onto a PVDF membrane (Millipore Corporation, Billerica, MA, USA). The membrane was incubated with primary antibodies and corresponding secondary antibodies. Protein bands were scanned using the chemiluminescence imaging system (GE Healthcare, Bucks, UK).

### Real-time PCR

Total RNA was extracted from cells using the Promega reagent (Promega, Beijing, China) according to the manufacturer’s instructions. Promega-A3500 kit was used for reverse transcription of RNA and quantitation of cDNA. The 2^-ΔΔCt^ method was used to calculate the relative expression level of target genes normalized to GAPDH expression.

Primers used in real time-PCR:

**Table:** 

Gene	Forward Primer	Reverse Primer
TXNIP	GATACCCCAGAAGCTCCTCC	ACCTCA GTGTAAGTGGGTGG
NLRP3	AGCCTTCCAGGATCCTCTTC	CTTGGGCAGCAGTTTCTTTC
Caspase-1	TGGCAGGAATTCTGGAGCTT	CTTGAGGGTCCCAGTCAGTC
IL-1β	CTG CTT CCA AAC CTT TGA CC	AGC TTC TCC ACA GCC ACA AT
IL-18	AAGACTCTTGCGTCAACTTCAAGGA	AGTCGGCCAAAGTTGTCTGATTC
GAPDH	TGATGGGTGTGAACCACGAG	AGTGATGGCATGGACTGTGG

### Elisa

At the end of each treatment described under section 2.2, the culture medium was collected, and the concentrations of IL-1β and IL-18 were measured using ELISA kits (MultiSciences Biotechnology, Hangzhou, China); manufacturer’s instructions were followed. The absorbance of each well was measured on a microplate reader (BioTek) using the wavelength of 450 nm. Each determination was performed in triplicate, the values were averaged, and the cytokine content was calculated using standard curves.

### Cer detection by immunofluorescence

At the end of the treatment (see section 2.2), the cells were fixed, permeabilized, and incubated with the anti-Cer antibody (1:500, ENZO, Switzerland) at 4 °C for 12 h. Subsequently, the cells were incubated with the secondary antibody in the dark at room temperature for 1 h. After washing, cells were stained with DAPI in the dark for 10 min. Nikon Eclipse 90i Fluorescence Microscope system (Nikon, Japan) was used to visualize the cells and record the images. The quantification of Cer in THP-1 macrophages utilized the diacylglycerol (DAG) kinase assay, as mentioned earlier [19].

### ASM activity measurement

The cells were lysed using a 1X mammalian lysis buffer, and the samples were treated with ASM assay reagents (ASM assay kit, Abcam, San Francisco, CA, USA) according to the manufacturer’s instructions. After incubating for 1 h at room temperature, fluorescence was detected using a microplate reader (BioTek) at the excitation and emission wavelength of 540 and 590 nm, respectively. The fluorescence in the blank wells was used as a negative control.

### NF-κB-p65 DNA-binding activity

The activation of the NF-κB complex was determined using an ELISA-based p65 transcription factor assay (Cayman Chemicals, Ann Arbor, MI, USA). The nuclear extract was fractionated using the extraction reagent kit (Thermo Fisher Scientific Inc., Rockford, USA) according to the manufacturer’s protocol. Oligonucleotides containing p65 uniform binding sites were immobilized in the wells and incubated with nuclear extracts. Finally, after incubation with specific primary and horseradish peroxidase (HRP)-conjugated secondary antibodies, the absorbance was measured at 450 nm.

### Statistical analysis

All experimental data are presented as the mean ± SD, and statistical analysis was conducted using SPSS version 23.0. The statistical significance of differences between the groups was determined using a one-way analysis of variance (ANOVA). *P* < 0.05 was considered to indicated statistical significance.

## Results

### Effects of imipramine, C2-Cer, and verapamil on cell viability

**Fig. 1:**
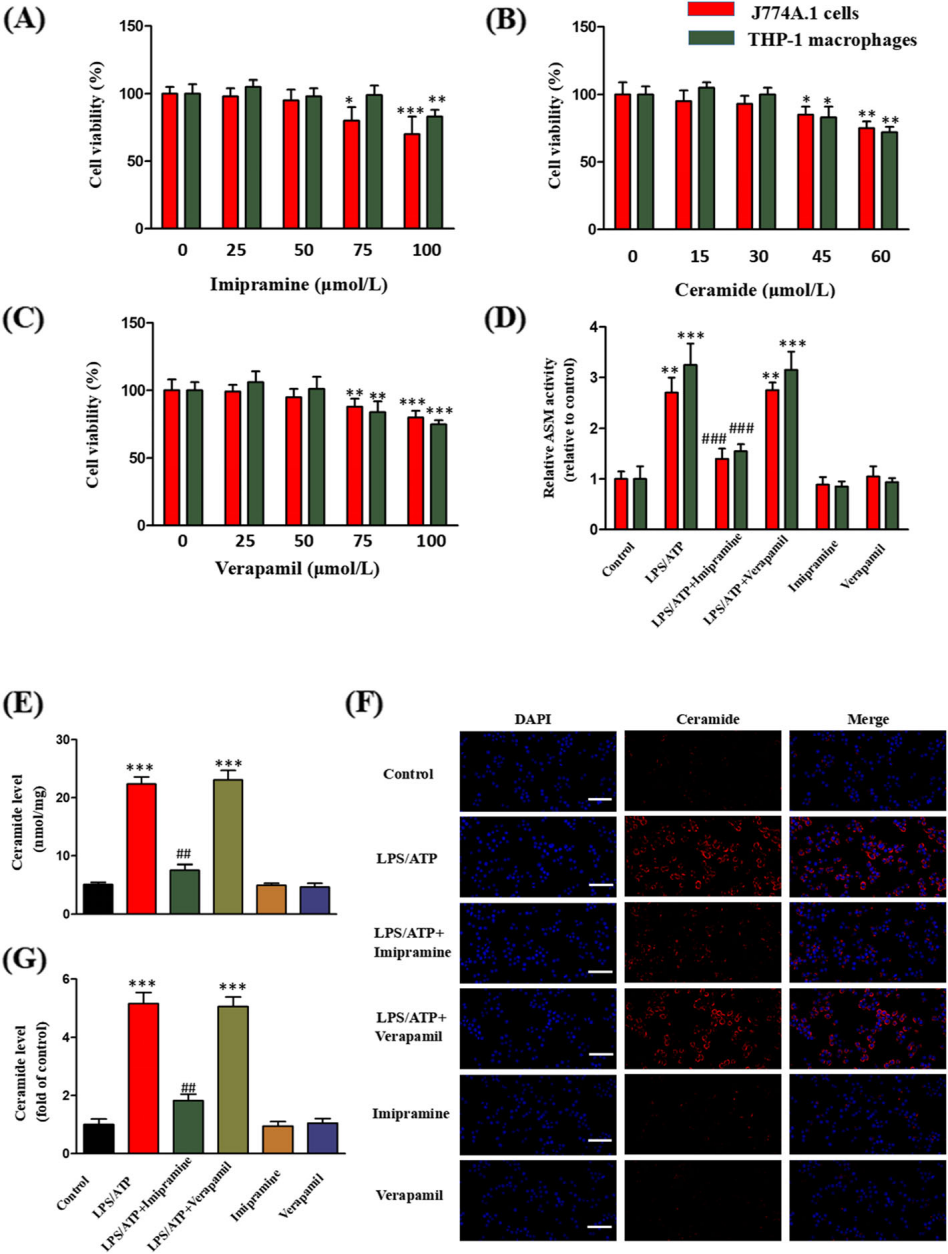
Effects of imipramine and verapamil on LPS/ATP-induced ASM activity and Cer production. The cells were treated with imipramine (0, 25, 50, 75, 100 μmol/L), C2-Cer (0, 15, 30, 45, 60 μmol/L) and verapamil (0, 25, 50, 75, 100 μmol/L) for 24 h. **a** Imipramine; **b** C2-Cer; **c** Verapamil. The J774A.1 cells and THP-1 macrophages were incubated with LPS (1 μg/mL) for 4 h, treated with imipramine or verapamil (10 μmol/L) for 3 h, and finally with ATP (5 mM) for 30 min. **d**. Effects of imipramine and verapamil on ASM activity in J774A.1 cells and THP-1 macrophages. **e** Effects of imipramine and verapamil on Cer formation in THP-1 macrophages. **f**, **g** Effects of imipramine and verapamil on Cer formation in J774A.1 cells. Bar = 50 μm. *P < 0.05, **P < 0.01, ***P < 0.001 vs. control group; ^##^*P* < 0.01, ^###^*P* < 0.001 vs. LPS/ATP treatment group

The effects of imipramine (0, 25, 50, 75, 100 μmol/L), C2-Cer (0, 15, 30, 45, 60 μmol/L), and verapamil (0, 25, 50, 75, 100 μmol/L) on the viability of J774A.1 cells and THP-1 macrophages were measured by the MTT method. Imipramine did not exert significant toxicity in J774A.1 cells at 0–50 μmol/L and in THP-1 macrophages at 0–75 μmol/L (Fig. 1a). C2-Cer at concentrations above 30 μmol/L decreased the viability of both types of cells (Fig. 1b). Verapamil at concentrations up to 50 μmol/L did not affect the viability of cells (Fig. 1c). Base on these results, the concentrations of 10 μmol/L of imipramine, 30 μmol/L of C2-Cer, and 10 μmol/L of verapamil were used in subsequent experiments.

### Imipramine pretreatment inhibits LPS/ATP-induced ASM activity and Cer production

To unravel the role of the ASM/Cer pathway in TXNIP/NLRP3 inflammasome activation, the effect of imipramine, an ASM activity inhibitor, on LPS/ATP-induced ASM activation and Cer generation was examined in J774A.1 cells and THP-1 macrophages. LPS/ATP significantly increased ASM activity, but this effect was inhibited by imipramine (Fig. 1d). Similarly, LPS/ATP significantly elevated the Cer content, and this effect was blocked by imipramine (Fig. 1e, f, g).

### Inhibition of ASM activity attenuates the LPS/ATP-induced TXNIP expression and NLRP3 inflammasome activation in J774A.1 cells and THP-1 macrophages

**Fig. 2:**
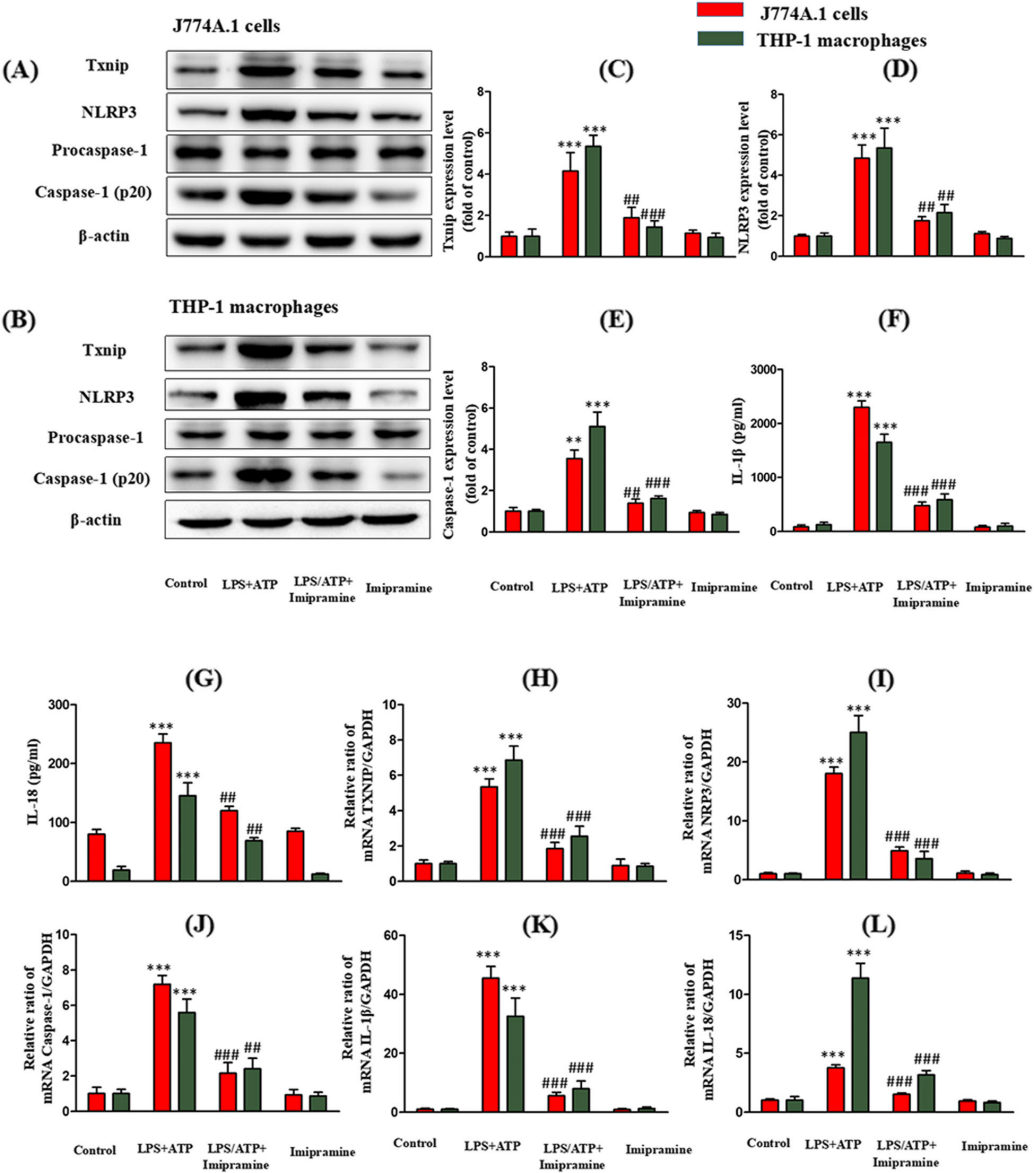
Inhibition of ASM activity attenuates LPS/ATP-induced TXNIP expression and NLRP3 inflammasome activation. The J774A.1 cells and THP-1 macrophages were incubated with LPS (1 μg/mL) for 4 h, treated with imipramine (10 μmol/L) for 3 h, and finally with ATP (5 mM) for 30 min. Effects of imipramine on the protein expression: **c** TXNIP; **d** NLRP3; **e** Caspase-1; **f** IL-1β; **g** IL-18. Effects of imipramine on the mRNA expression: **h** TXNIP; **i** NLRP3; **j** Caspase-1; **k** IL-1β; **l** IL-18. **P < 0.01, ***P < 0.001 vs. control group; ^##^ *P* < 0.01, ^###^*P* < 0.001 vs. LPS/ATP treatment group

Next, the effect of imipramine on the LPS/ATP-induced expression of TXNIP protein and the activation of NLRP3 inflammasome was determined. Imipramine suppressed the LPS/ATP-induced expression of TXNIP, NLRP3, and caspase-1 (Fig. 2a-e). NLRP3 inflammasome-induced expression of IL-1β and IL-18 was then evaluated to unravel the inhibitory effect of imipramine on NLRP3 inflammasome activation. In this experiment, the upregulation of NLRP3 by LPS/ATP significantly enhanced IL-1β and IL-18 secretion by the cells, and this increase was significantly inhibited by pretreatment with imipramine (Fig. 2f, g).

### Inhibition of ASM activity attenuates LPS/ATP-induced NLRP3 inflammasome and TXNIP mRNA expression in J774A.1 cells and THP-1 macrophages

The levels of the TXNIP, NLRP3, caspase-1, IL-1β, and IL-18 mRNA were significantly increased after treatment with LPS/ATP (Fig. 2h-l). Imipramine significantly inhibited the upregulation of these mRNAs.

### Inhibition of TXNIP attenuates LPS/ATP-induced NLRP3 inflammasome activation in J774A.1 cells and THP-1 macrophages

**Fig. 3:**
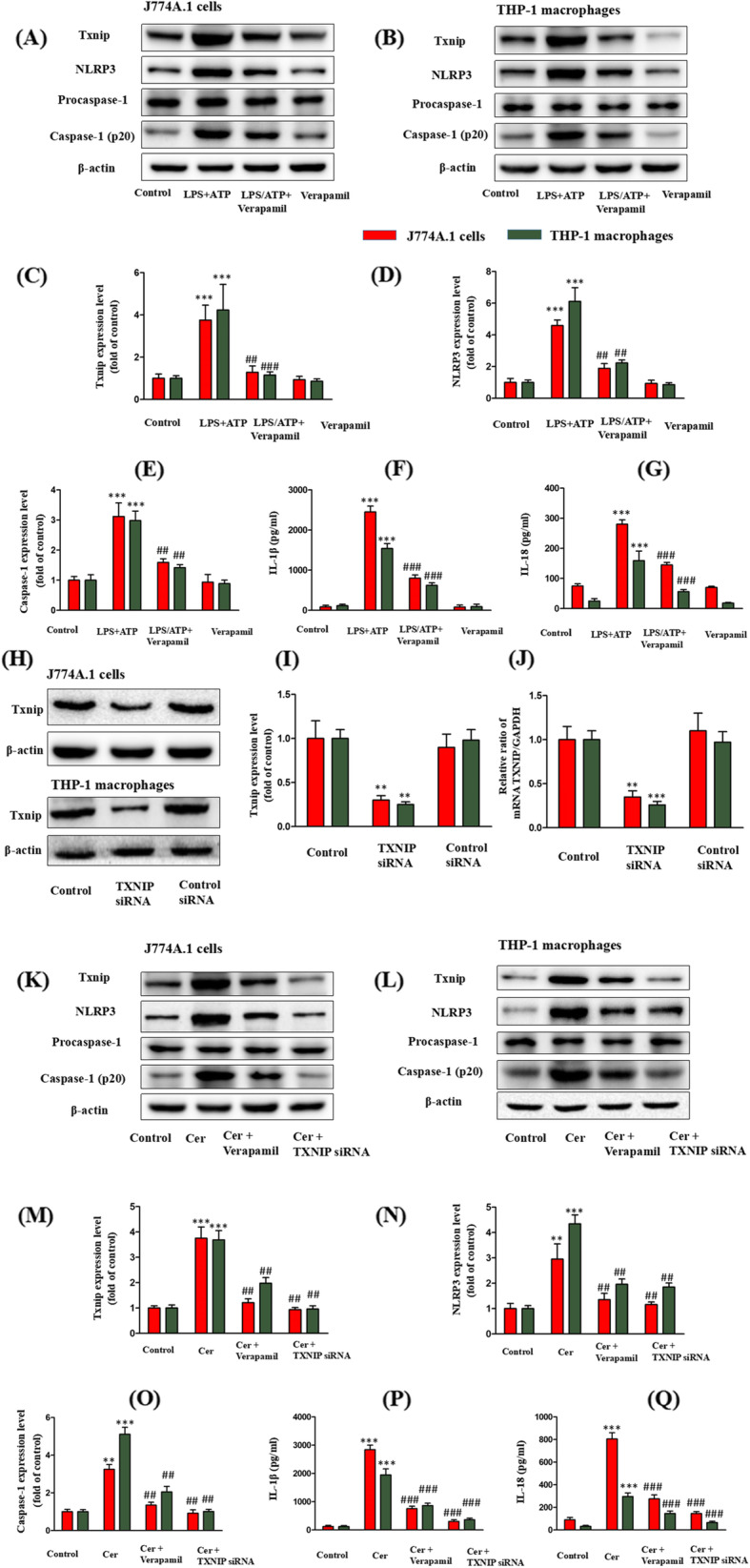
Inhibition of TXNIP attenuates LPS/ATP- or Cer-induced TXNIP expression and NLRP3 inflammasome activation. The J774A.1 cells and THP-1 macrophages were incubated with LPS (1 μg/mL) for 4 h, treated with verapamil (10 μmol/L) for 3 h, and finally with ATP (5 mM) for 30 min. Effects of verapamil on the protein expression: **c** TXNIP; **d** NLRP3; **e** Caspase-1; **f** IL-1β; **g** IL-18. The J774A.1 cells and THP-1 macrophages were pretreated with verapamil (10 μmol/L) for 3 h or TXNIP siRNA and then incubated with C2-Cer (30 μmol/L) for 5 h. Effects of siRNA on the expression of TXNIP: **i** TXNIP protein; **j** TXNIP mRNA. Effects of verapamil or TXNIP siRNA on the protein expression: **m** TXNIP; **n** NLRP3; **o** Caspase-1; **p** IL-1β; **q** IL-18. **P < 0.01, ***P < 0.001 vs. control group; ^##^*P* < 0.01, ^###^*P* < 0.001 vs. LPS/ATP treatment group or C2-Cer treatment group

Effects of verapamil (TXNIP inhibitor) on LPS/ATP-induced TXNIP expression level and NLRP3 inflammasome activation were evaluated next. LPS/ATP upregulated the expression of TXNIP, NLRP3, caspase-1, IL-1β, and IL-18, and this effect was mitigated by verapamil (Fig. 3a-g). The impact of verapamil on LPS/ATP-induced ASM activity and Cer content was also examined. Verapamil did not affect LPS/ATP-induced ASM activity and Cer content (Fig. 1d-f).

### Inhibition of TXNIP expression attenuates the Cer-induced NLRP3 inflammasome activation in J774A.1 cells and THP-1 macrophages

To determine whether TXNIP/NLRP3 inflammasome is downstream of the ASM/Cer pathway, the effect of verapamil or TXNIP siRNA on C2-Cer induced TXNIP expression and NLRP3 inflammasome activation was examined. siRNA effectively silenced TXNIP target genes (Fig. 3h-j). As expected, C2-Cer upregulated the expression of TXNIP, NLRP3, and caspase-1 protein, and verapamil or TXNIP siRNA suppressed this response (Fig. 3k-o). The effect of verapamil or TXNIP siRNA on C2-Cer-induced secretion of IL-1β and IL-18 was also evaluated. C2-Cer significantly elevated their secretion; however, this effect was significantly inhibited by pretreatment with verapamil or TXNIP siRNA (Fig. 3p, q).

### Inhibition of NF-κB attenuates Cer-induced TXNIP expression and NLRP3 inflammasome activation in J774A.1 cells and THP-1 macrophages

**Fig. 4:**
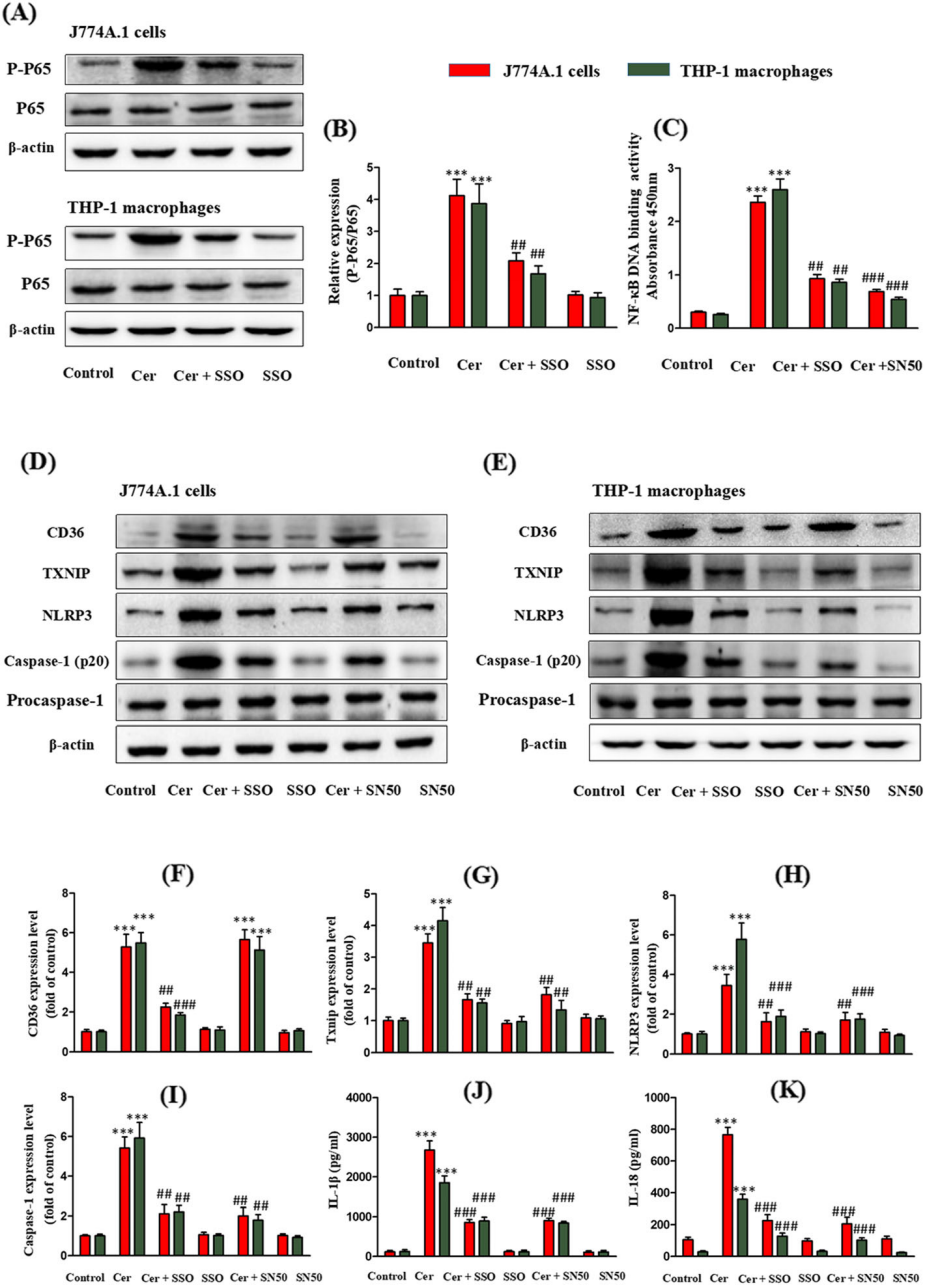
Inhibition of CD36 attenuates Cer-induced NF-κB activation and prevents subsequent activation of TXNIP/NLRP3 inflammasomes. The J774A.1 cells and THP-1 macrophages were pretreated with SSO (200 μmol/L) or NF-κB inhibitor SN50 (36 μmol/L) 1 h, and then incubated with C2-Cer (30 μmol/L for 5 h). **b** Effect of SSO on the activation of phospho-p65. **c** Effect of SSO and NF-κB inhibitor SN50 on Cer-induced p-P65 DNA-binding activity. Effect of SSO and NF-κB inhibitor SN50 on Cer-induced CD36 expression, TXNIP expression and NLRP3 inflammasomes: **f** CD36; **g** TXNIP; **h** NLRP3; **i** Caspase-1; **j** IL-1β; **k** IL-18. **P < 0.01, ***P < 0.001 vs. control group; ^##^*P* < 0.01, ^###^*P* < 0.001 vs. C2-Cer treatment group

We hypothesized that the Cer-induced up-regulation of TXNIP is related to the activation of NF-κB. NF-κB is a critical nuclear transcription factor regulating inflammation and cell survival. As expected, Cer increased the level of phospho-NF-κB-p65 and increased its binding to DNA in J774A.1 cells and THP-1 macrophages (Fig. 4a-c). However, the NF-κB inhibitor SN5O significantly reduced the NF-κB DNA binding activity in Cer-treated cells (Fig. 4c). At the same time, SN5O also significantly inhibited the expression of TXNIP and the activation of NLRP3 inflammasome induced by Cer (Fig. 4d-k).

### Inhibition of CD36 attenuates Cer-induced NF-κB activation and prevents subsequent activation of TXNIP/NLRP3 inflammasomes in J774A.1 cells and THP-1 macrophages

The irreversible inhibitor of CD36, sulfo-N-succinimidyl oleate (SSO), inhibited Cer-induced CD36 expression and significantly reduced the increase of phospho-NF-κB-p65 and the NF-κB DNA binding activity in Cer-treated cells (Fig. 4a-f). Therefore, we hypothesized that SSO blocks the Cer-stimulated expression of TXNIP. As expected, pretreatment of cells with SSO significantly reduced the expression of TXNIP and the activation of NLRP3 inflammasome (Fig. 4d-k). Taken together, these results suggest that Cer-mediated TXNIP/NLRP3 activation is regulated by the CD36/NF-κB pathway.

## Discussion

Macrophages, a vital part of the immune system, play a vital role in the innate immune response [20]. LPS is a crucial component of the extracellular membrane of gram-negative bacteria; it maintains the structural integrity of the cell and elicits pathogen-induced inflammation [21]. The LPS/ATP-stimulated J774A.1 cells and THP-1 macrophages represent a widely accepted in vitro model of inflammation used in the studies on the mechanism of NLRP3 inflammasome activation [22, 23]. Hence, this model was employed in the current study. Accumulating evidence indicates that the aberrant activation or dysregulation of NLRP3 inflammasome is manifested in the most prevalent inflammatory conditions [4]. An in-depth investigation of the signaling pathway implicated in the NLRP3 inflammasome activation could lead to the identification of more relevant drug targets for the effective management of inflammatory disorders. This study demonstrated the involvement of ASM in LPS/ATP-induced Cer production in macrophages, which activated the NLRP3 inflammasome by upregulating TXNIP expression. The obtained results further confirm that the CD36-dependent NF-κB-TXNIP signaling pathway plays an important role in the Cer-induced activation of NLRP3 inflammasomes in macrophages.

Sphingolipid metabolism is a significant part of lipid metabolism. It generates an array of active cellular lipids, imparts structural integrity to the cell, and regulates many crucial cellular functions [24, 25]. A plethora of stress stimuli trigger an excessive formation of Cer through sphingolipid metabolism. As a secondary signaling molecule, Cer activates signaling pathways required to initiate biological processes such as inflammation, apoptosis, and cellular differentiation [26, 27]. Accumulating evidence has demonstrated that Cer accumulation triggers NLRP3 inflammasome activation in various pathological conditions [7, 8]. The intracellular accumulation of Cer occurs through two significant routes of synthesis: de novo synthesis via ceramide synthase serine palmitoyltransferase (SPT) and hydrolysis of the membrane sphingomyelin by neutral sphingomyelinase (NSM)/ASM [26, 28]. However, recent studies suggested that the elevated level of Cer in stress response is an outcome of ASM-mediated sphingomyelin hydrolysis [9, 10]. Our previous findings have demonstrated that imipramine participates in the amelioration of LPS-induced pulmonary inflammation in mice by suppressing the level of Cer [14]. Imipramine is a well-known ASM inhibitor, which disrupts the interaction between ASM and lysosomal membrane, eliciting the destruction of ASM by lysosomes [29].

In this study, LPS/ATP treatment significantly elevated the ASM activity and the Cer level, and these changes were significantly attenuated by the ASM inhibitor imipramine. This finding implies the presence of a positive correlation between the LPS/ATP-induced Cer accumulation and ASM activation in J774A.1 cells and THP-1 macrophages. Additionally, imipramine suppressed LPS/ATP-induced activation of TXNIP/NLRP3 inflammasome. TXNIP contributes significantly to biological processes, such as inflammation, oxidative stress, cell apoptosis, and glucose and lipid metabolism [30]. These processes, which are major contributors to the onset of inflammatory disorders, are negatively impacted by the elevated level of TXNIP [31, 32]. It was previously shown that TXNIP serves the crucial task of NLRP3 assembly by directly interacting with NLRP3 [15, 32]. However, its role in ASM/Cer-mediated TXNIP/NLRP3 inflammasome activation remains unexplored, prompting us to investigate the involvement of the ASM/Cer signaling pathway as an upstream regulator of TXNIP/NLRP3 inflammasome activation. The TXNIP inhibitor verapamil has been widely employed in the management of inflammation to suppress TXNIP expression and the associated NLRP3 inflammasome activation [33]. In the current study, verapamil suppressed the LPS/ATP-induced TXNIP/NLRP3 inflammasome activation. However, verapamil did not affect LPS/ATP-induced activation of ASM and production of Cer. Therefore, J774A.1 cells and THP-1 macrophages were stimulated with C2-Cer to establish whether the TXNIP/NLRP3 inflammasome activation is a downstream event of the ASM/Cer signaling pathway. This experiment showed that C2-Cer significantly increased the expression of TXNIP, NLRP3, and caspase-1 proteins, and the secretion of IL-1β and IL-18. Furthermore, verapamil or TXNIP siRNA inhibited C2-Cer-mediated upregulation of TXNIP and activation of the NLRP3 inflammasome, suggesting that Cer induces TXNIP overexpression and subsequent NLRP3 inflammasome activation. Given that a high expression of TXNIP is associated with multiple pathological processes, further research is needed to identify the molecules that affect the regulation of TXNIP expression by Cer. The recognition of these molecules may lead to the identification of new drug targets. In this context, it is interesting that the Cer-induced activation of NF-κB is accompanied by an increase in TXNIP expression [34].

Recent results indicate that the NF-κB pathway is also involved in the regulation of NLRP3 inflammasome activation [35]. Cer is an effective inducer of the inflammatory response by activating NF-κB [35, 36]. However, the pathogenic role of the relationship among the NF-κB signaling pathway, TXNIP, and NLRP3 during Cer-induced NLRP3 inflammasome activation has not been fully understood. Of relevance, the NF-κB inhibitor SN-50 significantly inhibits Cer-induced NF-κB activation, TXNIP expression, and NLRP3 inflammasome activation. Therefore, it can be concluded that the activation of NF-κB is a key factor in the induction of TXNIP expression and NLRP3 inflammasome activation by Cer in macrophages. Previous studies have shown that Cer increases the expression of CD36 and that CD36 has an essential function in the activation of NLRP3 inflammasomes in macrophages [34, 37]. The current work demonstrated that Cer increases the expression of CD36 in macrophages, and the inhibition of CD36 by SSO significantly reduced Cer-induced TXNIP expression and NLRP3 inflammasome activation. Interestingly, the inhibitory effect of SSO on CD36 also significantly reduced the Cer-induced activation of NF-κB, which is upstream of TXNIP, but the inhibition of NF-κB activation did not affect the expression of CD36. In agreement with our findings, the activation level of NF-κB was significantly reduced in macrophages from CD36-deficient patients and CD36 knock-out mice [38, 39]. In addition, the activation of NF-κB and the release of IL-1β and TNF-α in CD36-deficient macrophages were significantly reduced [40]. In summary, these results reveal the critical role of the activation of the CD36-NF-κB signaling pathway in Cer-induced TXNIP expression and NLRP3 inflammasome activation.

### Study strengths and limitations

The advantages of this study are as follows. Firstly, the experiments documented that LPS/ATP induces Cer accumulation in macrophages by activating ASM, leading to subsequent activation of NLRP3 inflammasomes. Secondly, the study identified the involvement of the ASM/Cer/TXNIP signaling pathway in NLRP3 inflammasome activation. Thirdly, the CD36-dependent NF-κB/TXNIP signaling pathway was found to be essential for the Cer-induced activation of NLRP3 inflammasomes in macrophages. However, this study also has certain limitations. The results were derived from cell experiments in vitro, necessitating their verification by animal experiments in vivo.

## Conclusions

In conclusion, this study demonstrated the crucial role of the ASM/Cer/TXNIP signaling pathway in NLRP3 inflammasome activation. The important function of the CD36-NF-κB signaling pathway in the Cer-induced upregulation of TXNIP and subsequent activation of NLRP3 inflammasome was also shown. Further understanding of the mechanism of NLRP3 inflammasome activation might reveal novel approaches to the treatment of inflammatory diseases, such as acute lung injury and glomerular injury. The targeted regulation of the Cer signaling pathway may provide innovative therapeutic strategies for inflammatory diseases.

## Data Availability

Data are available from the authors upon request.

## Abbreviations

ASM: Acid sphingomyelinase
ATP: Adenosine triphosphate
Cer: Ceramide
ELISA: Enzyme-linked immunosorbent assay
DAG: Diacylglycerol
DMSO: Dimethylsulfoxide
HRP: Horseradish peroxidase
IL-18: Interleukin-18
IL-1β: Interleukin-1β
LPS: Lipopolysaccharide
NF-κB: Nuclear factor-κB
NLRP3: NOD-like receptor protein 3
NSM: Neutral sphingomyelinase
PMA: Phorbol 12-myristate 13-acetate
RT-PCR: Real-time PCR
SPT: Serine palmitoyltransferase
SSO: Sulfo-N-succinimidyl oleate
TXNIP: Thioredoxin interacting protein
TNF-α: Tumor necrosis factor-α
